# Supplementary material for: Brain derived neurotrophic factor declines after complete curative resection in gastrointestinal cancer
Source: PeerJ. 2021 Jul 30;9:e11718. doi: 10.7717/peerj.11718 (PMC8327966; doi:10.7717/peerj.11718)
Supplement: Supplemental Information 2 [file peerj-09-11718-s002.docx]

Codebook of BDNF raw data

B column:

Sex

Woman=1

Man=2

C column:

ICD 10 classification

1 colon cancer

2 pancreatic cancer

3 inguinal hernia

D column:

resection=1

laparotomy without resection=2

E column:

BDNF preop.=BDNF serum concentration before operation

F column:

BDNF postop.=BDNF serum concentration on the 3^rd^ day after operation

G, H, I columns:

TNM staging

J column:

R classification

R0=resection for cure or complete remission

R1=microscopic residual tumor

R2=macroscopic residual tumor.

Q:

G the [*grade*](https://en.wikipedia.org/wiki/Grading_(tumors)) of the cancer cells

T:

Histopathology

Adenoca=adenocarcinoma

U:

Tumour size in cm

V:

Tumour volume in cm^3^
